# Postnatal Prediction of Gestational Age Using Newborn Fetal Hemoglobin Levels

**DOI:** 10.1016/j.ebiom.2016.11.032

**Published:** 2016-12-01

**Authors:** Kumanan Wilson, Steven Hawken, Malia S.Q. Murphy, Katherine M. Atkinson, Beth K. Potter, Ann Sprague, Mark Walker, Pranesh Chakraborty, Julian Little

**Affiliations:** a Clinical Epidemiology Program, Ottawa Hospital Research Institute, Ottawa, Ontario, Canada; b Department of Medicine, University of Ottawa, Ottawa, Ontario, Canada; c Institute of Clinical Evaluative Sciences, uOttawa Site, Ottawa, Ontario, Canada; d School of Epidemiology, Public Health and Preventive Medicine, University of Ottawa, Ottawa, Ontario, Canada; e Department of Public Health Sciences, Karolinska Institutet, Stockholm, Sweden; f Better Outcomes Registry & Network, Ottawa, Ontario, Canada; g Department of Obstetrics, Gynecology and Newborn Care, The Ottawa Hospital, Ottawa, Ontario, Canada; h Newborn Screening Ontario, Ottawa, Ontario, Canada; i Department of Pediatrics, Children's Hospital of Eastern Ontario, Ottawa, Ontario, Canada

**Keywords:** Preterm birth, Gestational age, Metabolomics, Newborn screening, Prediction modelling

## Abstract

**Introduction:**

In many parts of the developing world procurement of antenatal gestational age estimates is not possible, challenging provision of appropriate perinatal care. This study aimed to develop a model for postnatal gestational age estimation utilizing measures of the newborn hemoglobin levels and other metabolic analyte data derived from newborn blood spot samples.

**Methods:**

We conducted a retrospective cohort analysis of 159,215 infants born January 2012–December 2014 in Ontario, Canada. Multivariable linear and logistic regression analyses were used to evaluate the precision of developed models.

**Results:**

Models derived from a combination of hemoglobin ratios and birthweight were more precise at predicting gestational age (RMSE1·23 weeks) than models limited to birthweight (RMSE1·34). Models including birthweight, hemoglobin, TSH and 17-OHP levels were able to accurately estimate gestational age to ± 2 weeks in 95·3% of the cohort and discriminate ≤ 34 versus > 34 (c-statistic, 0·98). This model also performed well in small for gestational age infants (c-statistic, 0·998).

**Discussion:**

The development of a point-of-care mechanism to allow widespread implementation of postnatal gestational age prediction tools that make use of hemoglobin or non-mass spectromietry-derived metabolites could serve areas where antenatal gestational age dating is not routinely available.

## Highlights

• Mechanisms for postnatal gestational age estimation are required to guide care in low resource settings.
• Newborn fetal/adult hemoglobin ratio and other non-mass spectrometry derived data can be used to provide precise estimates of gestational age.
• Hemoglobin derived postnatal gestational age prediction models also performed comparatively well in small for gestational age infants.

Three research groups including our own have recently published on the development of postnatal gestational age prediction algorithms derived from newborn screening metabolic profiles. Expanded newborn screening practices relying on tandem mass spectrometry instrumentation are not common place in many low resource settings, thus limiting the utility of such prediction models. Newborn fetal and adult hemoglobin levels are known to vary by gestational age of birth, and may be derived by methods other than mass spectrometry. In this study we used a retrospective cohort study design to develop and validate the precision of postnatal gestational age prediction models derived from fetal and adult hemoglobin levels, and readily available perinatal characteristics obtained from the Better Outcomes Registry & Network and the Newborn Screening of Ontario program. Final models were able to accurately predict postnatal gestational age to within 2 weeks of true gestational age, with excellent precision to discriminate the gestational age of average and small for gestational age infants. We have built upon our existing postnatal gestational age prediction algorithm to demonstrate both the stand-alone and additive predictive potential of newborn hemoglobin levels to the model. Methods to predict gestational age based on newborn screening markers have the potential to provide accurate postnatal assessments of gestational age in settings where gold standard first trimester ultrasounds are limited.

## 1 Introduction

Preterm birth affects over 15 million newborns each year and is the leading cause of neonatal mortality and morbidity worldwide, complications from which are the leading cause of neonatal mortality, and contributes to 40% of all deaths under the age of five (Lawn et al., 2012, Nour, 2012). The burden of preterm birth is particularly high in resource-poor settings where major risk factors including infection, inadequate nutrition, and poor socioeconomic circumstances are common (Beck et al., 2010). Knowledge of gestational age at the time of birth is critical for population level surveillance, to guide postnatal care by facilitating identification of infants with immediate high-resource needs and guiding developmental assessments (Dosman et al., 2012, Dipietro and Allen, 1991, Bonhoeffer et al., 2006). Differentiation of infants born by preterm birth versus those who are small for gestational age is important to further distinguish infant medical requirements. Unfortunately, in many low-resource environments limited access to prenatal ultrasound dating services and poor recall of self-reported menstrual histories impair accurate and timely gestational age assessment (Rijken et al., 2009, The Partnership for Maternal, 2006).

We and others have recently developed prediction models based on routinely collected newborn metabolic screening profiles that provide accurate estimates of gestational age (Jelliffe-Pawlowski et al., 2015, Ryckman et al., 2015, Wilson et al., 2016). Many newborn screening analytes used to identify rare metabolic conditions may only be reliably ascertained using tandem mass spectrometry – technology requiring significant financial resources and technical expertise. Hemoglobin (Hb) screening for inherited blood disorders such as sickle cell disease and β-thalassemia includes measurement of fetal (HbF) and adult (HbA) Hb levels. HbF is the primary protein for oxygen transport in the developing fetus. Hemoglobin production naturally shifts with advancing gestation from HbF to HbA such that HbF reserves are typically depleted by six months of age (Bank, 2006, Stamatoyannopoulos, 2005), and while residual amounts of HbF continue to be synthesized in adult erythropoiesis, the majority of adults have < 1% HbF (Thein et al., 2009). Contrary to the majority of metabolic analytes used in newborn screening programs which are measured by mass spectrometry, Hb may be measured using less technically demanding approaches including high performance liquid chromatography (HPLC) or gel electrophoresis (Association of public health laboratories, 2015, Clarke and Higgins, 2000).

Given the known relationship between HbF, HbA and gestational age, we sought to examine the effectiveness of Hb ratios in predicting gestational age at birth. The utility of Hb levels in a gestational age prediction model was compared to pre-existing prediction models incorporating newborn screening metabolites. We also evaluated models incorporating thyroid stimulating hormone and 17-hydroxyprogesterone, other non-mass spectrometry derived analytes.

## 2 Materials and Methods

A retrospective cohort study design was used to evaluate the precision of postnatal gestational age prediction models derived from fetal and adult Hb levels, other newborn screening analyte data and readily available perinatal characteristics obtained from infants born in Ontario, Canada. The study was approved by the Ottawa Hospital Science Network Research Ethics Board (20140724-01H) and the Children's Hospital of Eastern Ontario Research Ethics Board (15/143X).

### 2.1 Data Sources

#### 2.1.1 The Better Outcomes Registry and Network (BORN)

An Ontario maternal child registry that includes a broad collection of prenatal and perinatal data obtained from clinics, hospitals, labs, and midwifery practice groups. As a secondary use, data within the BORN Information System (BIS) is available to researchers.

#### 2.1.2 Newborn Screening Ontario (NSO)

Using heel-prick samples drawn from infants, usually within the first 72 h after birth, NSO screens virtually all infants born in the province for over 40 analytes to identify 29 rare conditions including metabolic disorders, endocrine disorders, hemoglobinopathies, immune deficiencies and other genetic disorders. Available screening analytes include markers of fatty acid oxidation, protein metabolism, endocrine function, immune function, and quantitative fetal and adult Hb levels. A summary of the newborn screening markers included in this study is provided in Table 1.

All live births captured in BIS between January 2012 and December 2014 were eligible for inclusion in the analysis. From this cohort, infants whose gestational age was determined by 1st trimester gestational dating ultrasound (from prenatal screening records), and who had complete newborn screening data were included. Infants who were positive for any of the conditions screened for by NSO were excluded, as were infants whose newborn screening samples were found to be of unsatisfactory quality by the screening laboratory. Finally, only infants whose screening samples were collected within 48 h of birth were included in the final analysis cohort, as the majority of infants born in low-resource settings are likely to be discharged from hospital within this time period.

### 2.2 Analysis

Four models were developed using the processes described below to assess predictive utility of HbA and HbF values alone, and in concert with other newborn screening analytes: (1) birthweight alone, (2) combination of birthweight and HbF and HbA levels (3) combination of birthweight, hemoglobin levels, TSH, and 17-OHP (all non mass-spectrometry derived analytes) and (4) full model including birthweight, and all newborn screening analytes including hemoglobin levels. Sex and multiple birth (yes, no) were included in all models. All analyses were conducted using SAS 9.4 and R v3.1.2.

### 2.3 Database Partition for Modelling

We used the same data partitioning strategy described previously (Wilson et al., 2016). In brief, the newborn cohort was divided into three dataset subsamples; one for model development, one to independently validate the choice of terms included in the final model, and one to independently test performance of the final model. Randomization was achieved using a stratified random sample approach, with stratification by sex and gestational age in weeks to ensure the same incidence of increasingly preterm birth was preserved in all subset data. Subsamples were generated using PROC SURVEYSELECT in SAS 9.4. Specifically, the newborn sample sets were partitioned according to a 2:1:1 ratio, distributing prematurity status (term, ≥ 37 weeks; near-term, 33–36 weeks; very preterm, 28–32 weeks, and extremely preterm, < 28 weeks) and sex evenly to ensure balance across the 3 datasets. The final analytical dataset was partitioned as follows: model development (n = 79,620), validation (n = 39,785) and test (n = 39,810) samples.

### 2.4 Predictive Modelling

Predictive modelling was performed using a multivariable linear regression model of continuous gestational age in weeks versus newborn screening analytes, sex, multiple birth status (yes, no) and birthweight. Continuous analyte and birthweight values were modelled using restricted cubic splines with four knots placed at quintile cut-points; 20th, 40th, 60th and 80th percentiles. Fetal (F, F1) and adult (A) Hb levels were modelled as (F + F1)/(A + F + F1), referred to Hb ratio throughout the remainder of this article. For restricted models including only birth weight and/or Hb ratio, nine knots were placed at decile cut points.

A weighted regression approach was employed in order to compensate for the smaller sample size and thus contribution to parameter estimation of preterm infants. Infants with lower gestational ages were weighted more heavily in model development to ensure that the fit was driven by both term and preterm infants.

Model building was conducted using the model development sample. A forward step-wise variable selection procedure was conducted using the *Swartz Bayesian Criterion* to guide the selection of covariates retained in the final model. Pairwise interactions were evaluated as part of stepwise variable selection. For interactions to be included in the model, contributing main effects had to be in the model. When no more terms could enter or leave the model, the stepwise procedure was terminated, and mean square error (MSE) was calculated by fitting the model from each iteration of the stepwise procedure to the independent validation data subset. The model generating the lowest MSE among all stepwise models was selected as the final model. Final model performance was then evaluated using the third test data subset, which had no role in model fitting or validation. This process provided maximum protection from over-fitting. The relative predictive power and precision of progressively more complex models were formally compared using both likelihood ratio tests (LRT), as well as performance metrics such as the MSE and AUC. Performance of models in sensitivity analyses were compared descriptively.

### 2.5 Model Performance for Classification as ≤ 34 weeks or > 34 weeks Gestational Age

In the current analysis, logistic regression models were also developed to distinguish between dichotomous categories of preterm birth (< 37 versus ≥ 37 weeks; and ≤ 34 versus > 34 weeks). Thirty-seven weeks represents the distinction between pre-term and term birth. Thirty-four weeks gestational age is an important clinical threshold as it represents the lower limit of the late preterm period (Kugelman and Colin, 2013, Bakewell-Sachs, 2007). Predictors of gestational age identified in the multiple linear regressions were used as independent variables in logistic regressions. Logistic regressions were fit to the model development sample, and evaluated in the independent test dataset.

### 2.6 Sensitivity Analyses

Model performance in terms of root mean squared error (RMSE), absolute prediction within ± 1 week, c-statistic (area under receiver operator curve, AUC), and positive predictive value (PPV) was evaluated overall, and in small for gestational age infants (infants in the lowest decile of birthweight given gestational age, SGA10) as well as in those infants from multiple births to investigate whether model prediction varied in quality across these subgroups. Lastly, model performance was also compared in heterozygotic carriers of sickle (HbS) and other hemoglobinopathy alleles (HbC, D, E, F) versus non-carriers (homozygotic HbA). Infants with two disease alleles were excluded during cohort creation as screen positives (HbS/S, HbS/C, HbS/β-thal).

## 3 Results

### 3.1 Sample Characteristics

**Fig. 1:**
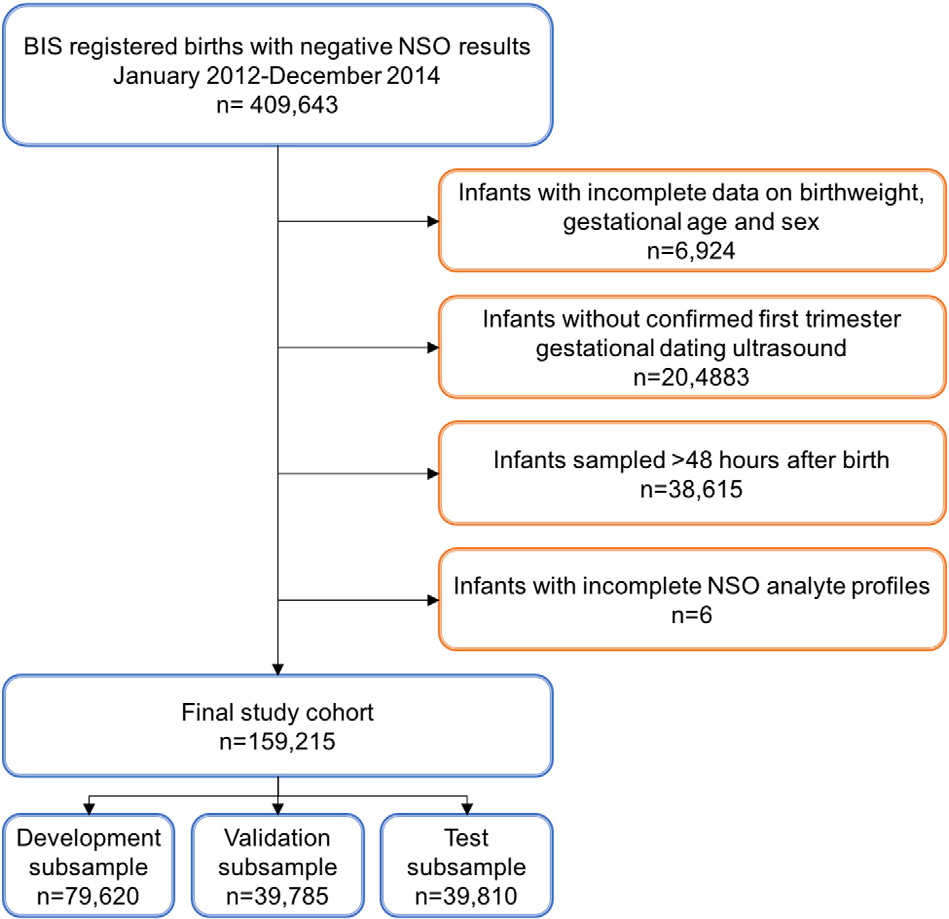
Cohort creation. Infants registered in the Born Information System (BIS) from January 2012 – December 2014 who were negative for the conditions screened by Newborn Screening Ontario (NSO) were used for analysis. Infants with incomplete essential demographic data or newborn screening profiles were excluded from the cohort, as were those without first trimester ultrasound data and those whose samples were collected > 48 h after birth.

**Fig. 2:**
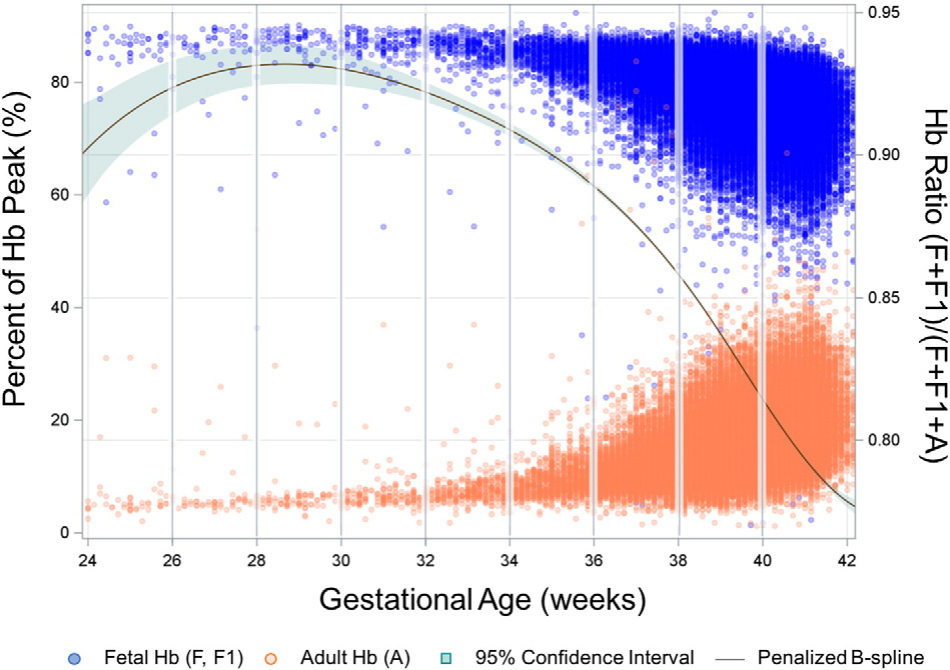
Distribution of fetal and adult hemoglobin levels by gestational age. Hb, hemoglobin.

Complete newborn screening records including all study analytes, sex and birth weight were available for 159,215 infants born between January 2012 and December 2014 (Fig. 1). A summary of the cohort characteristics is provided in Table 2. As expected, Hb ratio decreased with advancing gestational age at birth. Relative levels of HbF and HbA in infants born at varying gestational ages is represented in Fig. 2.

### 3.2 Overall Model Performance

Linear regression performance characteristics demonstrated that the model restricted to newborn birthweight, sex, and multiple birth status had an RMSE of 1·34 weeks in the overall cohort, and correctly classified the gestational age to ± 1 week in 55·2% of infants and to ± 2 weeks in 88·4% of infants. Addition of Hb ratio improved model performance with an RMSE of 1·23 weeks (LRT p < 0.0001), and accurately predicted gestational age to ± 1 or 2 weeks in 60·4% and 90·9% of the cohort, respectively. Model performance was further improved by addition of TSH and 17-OHP levels (RMSE 1·16 weeks, LRT p < 0.0001). In this model, gestational age was correctly classified to ± 1 week of 62·8% of infants, and to ± 2 weeks of 92·5% of infants. Optimal model performance was achieved by the full analyte model incorporating birthweight, sex, multiple birth status and all newborn screening analytes including Hb ratio. The full prediction model had an overall RMSE of 1·04 weeks, and was capable of providing accurate estimations of gestational age to ± 1 or ± 2 weeks of true gestational age in 68·7%, and 95·3% of the cohort, respectively. Consistent with our previous findings (Wilson et al., 2016), performance of all linear regression models was diminished in SGA10 infants. Comparison of linear regression model performance characteristics by gestational age is provided in Table 3. The proportions of infants correctly classified by gestational age are summarized in Table 4.

### 3.3 Model Performance in Dichotomous Pre-term Birth Categories

Dichotomization of preterm birth using thresholds of 34 or 37 weeks gestational age demonstrated assessed performance of all models to distinguish between preterm birth categories. By logistic regression, AUC and PPV at 80% sensitivity demonstrated robust model performance overall. For all models, performance was similar or more robust in SGA10 infants compared to the overall cohort (Table 5). Gestational age prediction models were more accurate at discriminating ≤ 34 versus > 34 weeks gestational age compared to < 37 versus ≥ 37 weeks gestational age. As with linear regression results, logistic regression prediction models derived from a combination of birthweight and Hb ratio had higher predictive capacity than models derived from birthweight alone. Inclusion of TSH and 17OHP levels produced better performance characteristics relative to the full analyte model for discriminating infants ≤ 34 versus > 34 weeks gestational age (AUC 0·981 vs 0·975; PPV at 80% sensitivity, 0·675 vs 0·53, LRT p < 0.0001). Limited Hb models (Hb ratio and Hb ratio + TSH + 17-OHP) better discriminated ≤ 34 versus > 34 weeks gestational age among SGA10 infants (AUC, all > 0·998 vs 0·997; PPV with 80% sensitivity 0·860 and 0·831 vs 0·710, respectively).

### 3.4 Sensitivity Analysis in Carriers of Disease-causing Hb Variants

Although infants who screened positive for hemoglobinopathies were excluded from our analysis, heterozygotic carriers of disease causing alleles without the disease phenotype were not excluded. In the test data where model performance was evaluated, there were 39,251 non-carriers, 515 sickle carriers, 44 other carriers (Table 6). The birthweight only model demonstrated little variation in model performance by Hb variant carrier status. Inclusion of Hb ratio to the model (alone, and with TSH and 17-OHP) improved performance in non-carriers (RMSE, 1·21 and 1.15 weeks, respectively), although it diminished model performance in sickle and other variant carriers. Similarly, for the full model including all measured analytes diminished model performance (RMSE 1·47 and 1·42 respectively for sickle and other carriers respectively vs 1·03 for non-carriers).

## 4 Discussion

Developing reliable methods for postnatal identification of gestational age dating are urgently required. In jurisdictions where access to ultrasound dating is of limited option, postnatal estimations would improve population surveillance in local areas to ultimately address issues of preterm birth prevention, and help target service delivery to high-risk mothers and preterm infants (Dosman et al., 2012, Dipietro and Allen, 1991, Bonhoeffer et al., 2006). Implementation of postnatal estimation tools would also directly benefit affected infants, guiding allocation of services necessary for improving outcome, including kangaroo mother care and appropriate respiratory management (World Health Organization, 2003). In this study, we have demonstrated that prediction models using relative fetal and adult Hb levels at birth, in combination with birthweight, sex and multiple birth data, can provide accurate postnatal gestational age estimation. The addition of other non-mass spectrometry derived newborn screening analytes, TSH and 17OHP, to multivariable regression models further improves their predictive power. Logistic regression analyses demonstrate that our hemoglobin-based prediction algorithms discriminate between ≤ 34 and > 34 weeks gestational age overall and in SGA10 infants with excellent precision.

The human β-globin locus on chromosome 11 houses ε-, γ-, δ- and β-globin genes that regulate human HbF and HbA expression. While the ε-globin gene is active in early fetal life, γ-globin genes are predominantly expressed for the production of HbF, (α_2_γ_2_) during the fetal period (Bank, 2006). The predominance of HbF during fetal life has been attributed to its increased oxygen affinity compared to other Hb variants. As pregnancy progresses, δ- and β-globin genes are activated, with the β-globin gene being the most highly expressed in adult erythrocytes. Indeed, Hb newborn screening levels for our cohort confirmed that Hb ratio, defined by Hb(F + F1)/Hb(A + F + F1), varies by gestational age at birth, with term infants exhibiting the lowest Hb ratios and increasingly preterm infants having a consistently higher Hb ratio.

There is considerable potential value in using metabolic markers as a measure of gestational age after birth. In particular, the use of Hb ratio in such a prediction model would provide substantial advantages over models derived from other traditionally screened analytes. Heel prick blood spot collection for expanded newborn screening is typically taken between 24 and 72 h after birth, the timing of which is critical for accurate interpretation of screening results. In low-resource settings, mothers and infants are often discharged within 48 h of birth, thus limiting the opportunity for connecting with infants after discharge. Hemoglobin analysis however is not limited to heel prick sampling and cord blood samples may be used reliably for analysis, providing results immediately after birth (Lobel et al., 1989). In addition, Hb measurements are traditionally taken by electrophoresis or HPLC in many laboratories (Association of public health laboratories, 2015, Clarke and Higgins, 2000), and thus are more amenable to measurement in settings where the equipment and expertise required for mass spectrometry dependent analyses may be limited. Commercially available field-portable HPLC systems are now available, and in time could be harnessed for newborn screening applications. Lastly, the prevalence of sickle cell traits and rates of hemoglobinopathies are high in nations of the middle east, northern Africa and south east Asia(Modell and Darlison, 2008, Piel et al., 2010, Williams and Weatherall, 2012). Penicillin prophylaxis for the first year of life is a simple, inexpensive treatment for infants affected by sickle cell disease who are at increased risk of life-threatening pneumococcal infections (Association of public health laboratories, 2015). Thus introduction of Hb testing in areas without established practices would provide dual benefits of gestational age prediction and identification of vulnerable children with hemoglobinopathy conditions.

The utility of newborn TSH and 17-OHP levels in addition to Hb ratios in model performance was also explored. Similar to hemoglobin, TSH and 17-OHP offer practical advantages as they may be routinely obtained by fluorometric or colorimetric assay rather than by mass spectrometry analysis. These analytes are also likely to be captured in existing newborn screening programs (Therrell et al., 2015) due to the frequency of related disorders and effectiveness of treatment. In our study, addition of TSH and 17-OHP improved model accuracy over and above that of the simple Hb model and demonstrated excellent predictive ability in SGA10 infants. The latter is particularly important in low resource settings where it may be difficult to distinguish infants who are small as a result of preterm birth or placental insufficiency. Although the effect of SGA10 on Hb ratio in the current study were not explored, preliminary examination of metabolic profiles derived from infants born to a tertiary care hospital with a diagnostic code of 'placental insufficiency' revealed no significant difference in Hb ratio. The disadvantages of relying on TSH and 17-OHP for gestational age prediction must also be considered. TSH and 17-OHP are subject to rapid postnatal change, and are thus typically sampled after analyte levels stabilize (Newborn Screening Ontario - Dépistage Néonatal Ontario, 2013). Thus it is unlikely that prediction models requiring such analyte measurements would be useful in infants who are discharged prior to 24 h, nor would the model be appropriate to cord blood-derived samples for the same reason.

While we found that models utilizing a full panel of newborn screening analytes were the most precise at predicting continuous GA than limited Hb models, the performance characteristics of our Hb models to discriminate ≥ 34 versus < 34 weeks gestational age are promising. Thirty-four weeks gestational age is an important clinical threshold as it represents the lower limit of the late preterm period (Kugelman and Colin, 2013, Bakewell-Sachs, 2007). It is above this threshold that the health risks of preterm infants are reduced, although still remaining elevated compared to their term counterparts (Nold et al., 2011). Thus, the trade-off between minor reduction in model accuracy with reduced cost and expertise required to obtain model variables may make hemoglobin-based metabolic prediction models for gestational age suitable substitutes to postnatal metabolic gestational dating in jurisdictions where full mass spectrometry screening is not available.

The strengths of these analyses include use of a large multi-ethnic cohort and our population-based approach. The large sample size enabled us to partition our data into independent derivation, test and validation sample sets, which permitted unbiased variable selection and avoided over fitting of the data. In addition, the use of gold standard first trimester ultrasound for gestational age strengthens the reliability of model performance. Potential limitations include the specificity of our model to the population from which it was derived. The majority of the infants included in model development were born at term, and average size for gestational age. Weighting of preterm and SGA infants in our model served to adjust for this, and we believe that a trade-off to favor accurate identification of infants at higher clinical risk is beneficial. Importantly, although derived from a multi-ethnic population, the performance of Hb models (Hb and birthweight alone, or the full model) in international populations is as of yet uncertain. Preliminary validation of our original gestational age model, however suggests robust performance across ethnic subgroup in the province of Ontario, Canada.

## 5 Conclusions

Methods to predict gestational age based on newborn screening markers are poised to provide accurate postnatal assessments of gestational age in settings where gold standard first trimester ultrasounds are limited. Here, we have built upon our existing postnatal gestational age prediction algorithm to demonstrate both the stand-alone and additive predictive potential of newborn Hb levels to the model. The clinical value of the prediction model limited to birthweight and Hb while excluding other newborn screening analytes would depend on the acceptability of the prediction error for gestational age. To further validate our Hb-based algorithm, an evaluation of the model in cord blood would be of benefit. Validation of our model in low-resource settings is also warranted to determine its utility in international settings, as global or region-specific hemoglobin-based algorithms may be of particular use in low resource settings where mass spectrometry analysis for traditionally screening markers are not available. The incremental benefits of this approach over standard gestational age assessment should also be evaluated in a low resource setting.

## Funding Sources

This study was funded by a Phase II Grand Challenges Exploration grant from the Bill & Melinda Gates Foundation (OPP1141535). The funding agency had no role in the study design, data collection, analyses, interpretation or writing of the report. No payments were received to write this article by a pharmaceutical company or other agency. No payments were received to write this article by a pharmaceutical company or other agency.

## Conflict of Interest

The authors report no conflict of interest.

## Author Contributions

Conceptualization, KW; Methodology, KW, SH; Validation, KW, SH; Formal Analysis, SH; Resources, AS, MW, PC; Data Curation, MM, KA; Writing – Original Draft, KW, MM; Writing – Review & Editing, KW, MM, SH, KA, BP, AS, MW, PC, JL; Visualization, KW, MM; Supervision, KW; Project Administration, MM, KA; Funding Acquisition, KW, SH.

## Figures and Tables

**Table 1:** Newborn screening markers included for predictive modelling.

Acyl-carnitines	C0; C2; C3; C4; C5; C5:1; C6; C8; C8:1; C10; C10:1; C12; C12:1; C14; C14:1; C14:2; C16; C18; C18:1; C18:2; C10:1; C12:1; C14:1; C14:2; C4OH; C5:1; C5DC; C5OH; C6DC; C16:OH; C16:1OH; C18OH; C18:1OH; C3DC; C4DC
Amino acids	Arginine; phenylalanine; alanine; leucine; ornithine; citruline; tyrosine; glycine; argininosuccinate; methionine; valine; succinylacetone
Hemoglobins	Adult hemoglobin: HbA(A) and variants (S, C, D, E) fetal hemoglobin: HbF (F), acetylated HbF (F1), combined HbF (F + F1)
Endocrine markers	17-Hydroxyprogesterone (17-OHP), thyroid stimulating hormone (TSH)
Enzyme markers	Biotinidase; galactose-1-phosphate uridyltransferase (GALT); immunotripsinogen

**Table 2:** Sample characteristics.

Variable	All n = 159,215	≥ 37 weeks n = 150,257	> 34 to < 37 weeks n = 7,315	≤ 34 weeks n = 1,643
Sex				
Male, n (%)	80,838 (50·7)	76,123 (50·7)	3,852 (52·7)	863 (52·5)
Female, n (%)	78,377 (49·2)	74,134 (49·3)	3,463 (47·3)	780 (47·5)
Gestational age, weeks mean (min-max)	39·3 (22·3–43·9)	39·5 (37·0–43·9)	36·0 (34·1–36·9)	31·3 (22·3–34·0)
Birth weight mean (min–max)	3374 (500–6210)	3426 (743–6210)	2682 (830–5605)	1736 (500–4500)
Small for gestational age, n (%)	15,845 (9·95)	14,005 (9·3)	796 (10·9)	139 (8·5)
Multiple births, n (%)	4412 (2·8)	2399 (1·60)	1491 (20·4)	522 (31·8)
Hb(F + F1)/Hb(A + F + F1) mean (min–max)	0·82 (0·05–1·00)	0·82 (0·05–1·00)	0·89 (0·11–0·97)	0·92 (0·40–0·98)

**Table 3:** Comparison of model performance overall and in SGA10 infants by gestational age category.

	All infants, RMSE				SGA10, RMSE			
Model^a^	Overall	≤ 34 weeks	34–37 weeks	≥ 37 weeks	Overall	≤ 34 weeks	34–37 weeks	≥ 37 weeks
Birthweight only	1·34	2·55	1·75	1·30	2·61	2·14	2·40	2·61
Birthweight + Hb ratio	1·23	2·58	1·45	1·19	2·23	2·22	2·01	2·23
Birthweight + Hb ratio + TSH + 17-OHP	1·16	2·44	1·35	1·13	1·94	1·35	1·57	1·94
Full model	1·04	2·24	1·16	1·01	1·52	1·33	1·26	1·52

RMSE, root mean square error (average absolute deviation of observed vs. predicted GA in weeks); TSH, thyroid stimulating hormone; 17-OHP, 17 hydroxyprogesterone; SGA10, small for gestational age.

a All models include infant sex, multiple birth (yes, no).

**Table 4:** Proportion of infants with gestational age correctly classified to ± 1 week and 2 weeks.

Model^a^		All infants, %				SGA10, %			
		Overall	≤ 34 weeks	34–37 weeks	≥ 37 weeks	Overall	≤ 34 weeks	34–37 weeks	≥ 37 weeks
Birthweight only	± 1 week	55·2	40·7	39·9	56·1	1·0	21·6	1·61	0·78
	± 2 week	88·4	72·0	71·7	89·4	36·6	54·1	44·6	36·0
Birthweight + Hb ratio	± 1 week	60·4	42·3	51·5	61·0	12·6	32·4	25·8	11·6
	± 2 week	90·9	71·5	83·8	91·4	53·1	51·4	61·8	52·6
Birthweight + Hb ratio + TSH + 17-OHP	± 1 week	62·8	47·6	55·8	63·3	23·7	51·4	37·1	22·6
	± 2 week	92·5	80·1	86·6	92·9	66·4	89·2	80·7	65·4
Full model	± 1 week	68·7	56·7	64·4	69·1	43·0	54·1	56·5	42·2
	± 2 week	95·3	86·1	91·4	95·6	83·7	89·2	88·7	83·4

RMSE, root mean square error (average absolute deviation of observed versus predicted GA in weeks); TSH, thyroid stimulating hormone; 17-OHP, 17 hydroxyprogesterone; SGA10, small for gestational age.

a All models include infant sex, multiple birth (yes, no).

**Table 5:** Performance of gestational age models to discriminate dichotomous categories of prematurity.

Model^a^	Preterm threshold	Overall		SGA10	
		AUC	PPV 80%	AUC	PPV 80%
Birthweight only	≤ 34 weeks	0·978	0·496	0·998	0·75
	< 37 weeks	0·901	0·240	0·983	0·627
Birthweight + Hb ratio	≤ 34 weeks	0·982	0·624	0·999	0·860
	< 37 weeks	0·931	0·334	0·973	0·546
Birthweight + Hb ratio + TSH + 17-OHP	≤ 34 weeks	0·981	0·675	0·998	0·831
	< 37 weeks	0·939	0·372	0·971	0·538
Full model	≤ 34 weeks	0·988	0·807	0·997	0·710
	< 37 weeks	0·957	0·476	0·970	0·510

PPV 80%, positive predictive value when the classification cutpoint is set such that sensitivity is 80%; AUC, area under the receiver operator curve (c-statistic); SGA10, small for gestational age; TSH, thyroid stimulating hormone; 17OHP, 17 hydroxyprogesterone.

a All models include infant sex, multiple birth (yes, no).

**Table 6:** Model performance by carriers of variant hemoglobins.

Model^a^	Overall, RMSE (n = 39,810)	Carrier status, RMSE		
		Non-carriers (n = 39,251)	Sickle carriers (n = 515)	Other carriers (n = 44)
Birthweight only	1·34	1·34	1·34	1·27
Birthweight + Hb ratio	1·23	1·21	1·95	2·05
Birthweight + Hb ratio + TSH + 17-OHP	1·16	1.15	1.86	2.09
Full model	1·04	1.03	1.47	1.42

TSH, thyroid stimulating hormone; 17OHP, 17-hydroxyprogesterone; RMSE, root mean squared error. Non-carrier, homozygotic HbA; Sickle Carrier, heterozygotic HbS; Other Carrier, heterozygotic HbC, D, E, F.

a All models include infant sex, multiple birth (yes, no).

## References

[bb0090] Association of public health laboratories (2015). Hemoglobinopathies: Current Practices for Screening, Confirmation and Follow-up. https://www.cdc.gov/ncbddd/sicklecell/documents/nbs_hemoglobinopathy-testing_122015.pdf.

[bb0005] Bakewell-Sachs S. (2007). Near-term/late preterm infants. Newborn Infant Nurs. Rev..

[bb0010] Bank A. (2006). Regulation of human fetal hemoglobin: new players, new complexities. Blood.

[bb0015] Beck S., Wojdyla D., Say L., Betran A.P., Merialdi M., Requejo J.H., Rubens C., Menon R., Van Look P.F. (2010). The worldwide incidence of preterm birth: a systematic review of maternal mortality and morbidity. Bull. World Health Organ..

[bb0020] Bonhoeffer J., Siegrist C.A., Heath P.T. (2006). Immunisation of premature infants. Arch. Dis. Child..

[bb0025] Clarke G.M., Higgins T.N. (2000). Laboratory investigation of hemoglobinopathies and thalassemias: review and update. Clin. Chem..

[bb0030] Dipietro J.A., Allen M.C. (1991). Estimation of gestational age: implications for developmental research. Child Dev..

[bb0035] Dosman C.F., Andrews D., Goulden K.J. (2012). Evidence-based milestone ages as a framework for developmental surveillance. Paediatr. Child Health.

[bb0040] Jelliffe-Pawlowski L.L., Norton M.E., Baer R.J., Santos N., Rutherford G.W. (2015). Gestational dating by metabolic profile at birth: a California cohort study. Am. J. Obstet. Gynecol..

[bb0045] Kugelman A., Colin A.A. (2013). Late preterm infants: near term but still in a critical developmental time period. Pediatrics.

[bb0050] Lawn J.E., Kinney M.V., Black R.E., Pitt C., Cousens S., Kerber K., Corbett E., Moran A.C., Morrissey C.S., Oestergaard M.Z. (2012). Newborn survival: a multi-country analysis of a decade of change. Health Policy Plan..

[bb0055] Lobel J.S., Cameron B.F., Johnson E., Smith D., Kalinyak K. (1989). Value of screening umbilical cord blood for hemoglobinopathy. Pediatrics.

[bb0060] Modell B., Darlison M. (2008). Global epidemiology of haemoglobin disorders and derived service indicators. Bull. World Health Organ..

[bb0065] Newborn Screening Ontario - Dépistage Néonatal Ontario (2013). Newborn Screening Manual: A Guide for Newborn Care Providers. https://www.newbornscreening.on.ca/sites/default/files/cho0095-nsm-pages-jan2015-web.pdf.

[bb0070] Nold C., Hussain N., Smith K., Campbell W., Borgida A., Egan J. (2011). Optimal time for delivery with preterm premature rupture of membranes from 32 to 36 6/7 weeks. J. Matern. Fetal Neonatal Med..

[bb0075] Nour N.M. (2012). Premature delivery and the millennium development goal. Rev. Obstet. Gynecol..

[bb0080] Piel F.B., Patil A.P., Howes R.E., Nyangiri O.A., Gething P.W., Williams T.N., Weatherall D.J., Hay S.I. (2010). Global distribution of the sickle cell gene and geographical confirmation of the malaria hypothesis. Nat. Commun..

[bb5000] Rijken M.J., Lee S.J., Boel M.E., Papageorghiou A.T., Visser G.H.A., Dwell S.L.M. (2009). Obstetric ultrasound scanning by local health workers in a refugee camp on the Thai-Burmese border. Ultrasound Obstet. Gynecol..

[bb0085] Ryckman K.K., Berberich S.L., Dagle J.M. (2015). Predicting gestational age by the use of neonatal metabolic markers. Am. J. Obstet. Gynecol..

[bb0095] Stamatoyannopoulos G. (2005). Control of globin gene expression during development and erythroid differentiation. Exp. Hematol..

[bb6000] The Partnership for Maternal (2006). Newborn and Child Health, Opportunities for Africa's newborns: practical data, policy and programmatic support for newborn care in Africa, Chapter 2: Antenatal Care. http://www.who.int/pmnch/media/publications/aonsectionIII_2.pdf.

[bb0100] Thein S.L., Menzel S., Lathrop M., Garner C. (2009). Control of fetal hemoglobin: new insights emerging from genomics and clinical implications. Hum. Mol. Genet..

[bb0105] Therrell B.L., Padilla C.D., Loeber J.G., Kneisser I., Saadallah A., Borrajo G.J., Adams J. (2015). Current status of newborn screening worldwide: 2015. Semin. Perinatol..

[bb0110] Williams T.N., Weatherall D.J. (2012). World distribution, population genetics, and health burden of the hemoglobinopathies. Cold Spring Harb. Perspect. Med..

[bb0115] Wilson K., Hawken S., Potter B.K., Chakraborty P., Walker M., Ducharme R., Little J. (2016). Accurate prediction of gestational age using newborn screening analyte data. Am. J. Obstet. Gynecol..

[bb7000] World Health Organization (2003). Kangaroo Mother Care: A Practical Guide. http://apps.who.int/iris/bitstream/10665/42587/1/9241590351.pdf.

